# Global land moisture trends: drier in dry and wetter in wet over land

**DOI:** 10.1038/srep18018

**Published:** 2015-12-11

**Authors:** Huihui Feng, Mingyang Zhang

**Affiliations:** 1 Key Laboratory of Watershed Geographic Sciences, Nanjing Institute of Geography and Limnology, Chinese Academy of Sciences, Nanjing, 21008, China; 2 Key Laboratory of Agro-ecological Processes in Subtropical Region, Institute of Subtropical Agriculture, Chinese Academy of Sciences, Changsha 410125, China

## Abstract

The “dry gets drier, wet gets wetter” (DGDWGW) paradigm is widely accepted in global moisture change. However, Greve *et al.*^1^ have declared that this paradigm has been overestimated. This controversy leaves a large gap in the understanding of the evolution of water-related processes. Here, we examine the global moisture trends using satellite soil moisture for the past 35 years (1979–2013). Our results support those of Greve *et al.*, although there are quantitative differences. Generally, approximately 30% of global land has experienced robust moisture trends (22.16% have become drier, and 7.14% have become wetter). Only 15.12% of the land areas have followed the DGDWGW paradigm, whereas 7.77% have experienced the opposite trend. A new finding is that there is a significant “drier in dry, wetter in wet” (DIDWIW) trend paradigm; 52.69% of the drying trend occurred in arid regions, and 48.34% of the wetter trend occurred in the humid regions. Overall, 51.63% of the trends followed the DIDWIW paradigm, and 26.93% followed the opposite trend. We also identified the DGDWGW and DIDWIW paradigms in low precipitation-induced arid regions in which the dry soil led to an increasing sensible heat flux and temperature and subsequently potential evapotranspiration.

Moisture has a crucial impact on the global climate, hydrology, ecology and environment. Currently, there is a rising contradictory issue regarding global moisture trends. Some researchers have warned of an increasing risk of drought under global warming^2^,^3^,^4^,^5^. However, it has been reported that global drought has been overestimated because of uncertainties and errors in the data sources and indices used^6^,^7^,^8^. Recently, the “dry gets drier, wet gets wetter” (DGDWGW) paradigm has become a common view^9^,^10^,^11^,^12^,^13^. However, writing in *Nature Geoscience*, Greve *et al.* 1 declared that aridity over the land has not followed a simple intensification of DGDWGW. Using a combination of hydrological data sets, they found that only 10.8% of the global land surface has presented a robust DGDWGW pattern compared with 9.5% with the opposite pattern. This controversy requires full clarification to understand global moisture changes and to support water use strategies.

Several factors have contributed to the controversy, primarily the various data sources that have been used in previous studies. Global moisture is commonly related to the hydrological variables of precipitation, evapotranspiration and soil moisture. The data are usually obtained from ground measurements, model simulations and satellite observations^14^,^15^. Specifically, ground measurements are a traditional and accurate method for measuring hydrological variables. However, ground measurements only represent a few square metres, and its application at a large scale is difficult. Model simulations estimate the variables by integrating atmospheric and environmental factors; however, their performances are usually weakened because complex physical processes in them are simplified. In recent decades, satellite remote sensing has been widely adopted to estimate the moisture data because of its spatially consistent view at a global scale. Using such satellite-derived data, various indices (i.e., the Palmer Drought Severity Index (PDSI)^2^,^16^, standardized precipitation index (SPI)^17^,^18^ and soil moisture percentile (SMP)^19^,^20^) have been derived to quantify moisture trends. The calculations of these indices depend on a series of assumptions that generate significant errors and uncertainties^7^,^8^,^21^. It would, therefore, be more reliable to monitor global moisture changes using hydrological variables rather than derived indices. Finally, the classification of climate regions unavoidably affects the spatial analysis of moisture trends. The simplest classification is to identify wet and dry regions as those with the highest and lowest precipitation^12^,^22^. Another common way is to define regions by coupling potential evaporation and precipitation^1^. Because of the different classification criteria, regions are usually with spatial disagreement.

Here, we examine global moisture trends using soil moisture because it connects precipitation and evapotranspiration in the water cycle process. The Climate Change Initiative (CCI) global soil moisture of the European Space Agency (ESA) rather than indices such as the PDSI was used for our analysis to reduce errors and uncertainties. A date set was produced by merging active and passive microwave satellite observations with a resolution of 25 km recorded from 1978 to 2013^23^,^24^,^25^. The accuracy is acceptable when validated by global ground-based observations, and the mean correlation coefficient (R) and root mean square error (RMSE) are 0.46 and 0.04 cm^3^•cm^−3^ 24, respectively. To analyse the spatial patterns of the trends, we first defined and classified the arid and humid regions using the updated Koppen-Geiger climate classification (see Methods)^26^,^27^. Then, we evaluated the spatial consistency between the global trends and the climate regions and finally quantified the agreement between the trends’ paradigms (see Methods).

Figure 1 presents the spatial pattern of global soil moisture. The multi-year mean is 0.227 ± 0.088 cm^3^•cm^−3^ over land. Globally, the dry soils are primarily located in southeast North America, North Africa, southwest Europe, central Asia and Australia. The wet areas are mainly located in northern North America, northern Europe and Southeast Asia. At the scale of climate regions, the soil moisture is 0.144 cm^3^•cm^−3^, 0.271 cm^3^•cm^−3^ and 0.245 cm^3^•cm^−3^ in the arid, humid and transitional regions, respectively. More specifically, the driest climate region (0.099 cm^3^•cm^−3^) is the hot desert, as represented by the Sahara desert in North Africa, whereas the wettest region (0.307 cm^3^•cm^−3^) occurs in the rainforest, as represented by the Amazon River Basin in Sorth America.

Figure 2 shows the temporal trends of global soil moisture, which present significant spatial variability. The percentage of the land that became drier was 22.16%, and 7.14% became wetter; the rates were −0.0023 cm^3^•cm^−3^•year^−1^ and 0.0029 cm^3^•cm^−3^•year^−1^, respectively. This result is consistent with that of Dorigo *et al.*^28^. Spatially, the drier areas were primarily located in northeastern North America, North Africa, central Asia and Southwest Australia. The wetter areas were primarily in Northwest Africa, northern South America, Northeast Europe and Northeast Asia. By comparing Figs 1 and 2, it can be seen that the drier trend was mainly confined to the dry regions, whereas the wetter trend was mainly confined to the wet regions.

Figure 3 shows the spatial consistency of the trends and climate regions. The percentages of the arid, humid and transitional regions that became drier were 38.41%, 16.34% and 12.99% respectively, and 2.91%, 8.05% and 10.46% of arid, humid and transitional regions, respectively, became wetter. The arid and humid climate regions cover 73.22% of the global land. However, only 15.12% of global land followed the DGDWGW paradigm, and 7.77% showed the opposite trend. This result demonstrates that the DGDWGW paradigm overestimates global moisture trends. Our results support that of Greve *et al.*^1^, although they are quantitatively different. Greve *et al.*^1^ showed that 10.8% and 9.5% of the land followed the paradigm and behaved opposite to the paradigm, respectively. This difference is because North Africa is absent in the analysis by Greve *et al.*^1^. North Africa’ has a topical arid climate and covers a large area. Our results show that “dry gets drier” is the most significant paradigm in this sub-region, with 59.59% of the area becoming drier. The absence of North Africa caused a large loss of information that would affect the trends that were reported in Greve at al.^1^. Although the DGDWGW is insignificant, there is an obvious paradigm that could be characterized as “drier in dry, wetter in wet” (DIDWIW) (see Methods). The percentage of the drying conditions that occurred in the arid, humid and transitional regions was 52.69%, 31.61% and 15.70%, respectively. Meanwhile, 12.41%, 48.34% and 39.25% of the increasing moisture conditions occurred in the arid, human and transitional regions, respectively. In total, 51.63% of the trends followed the DIDWIW paradigm, whereas 26.93% showed the opposite behaviour. The DIDWIW paradigm in the tropical sub-regions was also evaluated to validate this result. The largest sub-regions of the arid and humid climate regions (BWh and Dfc, respectively) were selected for evaluation. The BWh sub-region is characterized by a hot desert climate and covers 13.52% of the global land area. The area of increasing dryness in this sub-region was 36.36% of the area of increasing global dryness, which is approximately three times that of the area ratio over the land of the sub-region. The Dfc sub-region is characterized by a climate with cold summers and without a dry season. It covers 18.69% of the global land area and occupies 22.46% of the global wetter area. These results further confirm the observation that drier conditions were increasingly likely in arid regions, whereas increasing moisture were more likely occur in humid regions.

To capture the trends, we further identified the regions of the DGDWGW and DIDWIW paradigms. We first divided the trends in arid areas into “drier in dry” (DID) and “wetter in dry” (WID) regions and the trends in the humid areas into “wetter in wet” (WIW) and “drier in wet” (DIW) areas. Then, we compared the climate and moisture characteristics of the four areas. Using this method, it was easy to identify the background of the two paradigms. Precipitation and evapotranspiration are the two most important variables that affect soil moisture, which act to supply and deplete water in the soil, respectively, and were adopted for our analysis (the data sources are described in the Methods section)^29^,^30^. The statistical results show that the soil moisture, precipitation and evapotranspiration were 0.108 cm^3^•cm^−3^, 151.76 mm and 108.63 mm, respectively, in the DID, and 0.147 cm^3^•cm^−3^, 380.73 mm and 284.23 mm, respectively, in the WID. Regarding the trends in humid region, the three variables were 0.267 cm^3^•cm^−3^, 690.40 mm and 367.25 mm, respectively, in the WIW, and 0.269 cm^3^•cm^−3^, 713.84 mm and 375.01 mm, respectively, in the DIW. From the viewpoint of climate, these results suggest that the two drier paradigms can be identified in the low precipitation-induced arid areas. The main reason is that low precipitation led to soil moisture deficits in those areas. The dry soils then raised the sensible heat flux, which produced a warmer and drier low-level atmosphere and increased the potential evapotranspiration^31^. The minor differences in the WIW and DIW regions demonstrate that there are more complex hydrological processes in the humid regions^32^.

Overall, we conclude that the DGDWGW paradigm has been overestimated in previous studies. Alternatively, the trend presents a significant “drier in dry, wetter in wet” (DIDWIW) paradigm. The two paradigms offer two different concepts. The DGDWGW is evaluated at the scale of climate regions. DGDWGW suggests that the climate regions experiences drier or wetter over the land, which greatly overestimates the degree of global moisture trends. In fact, only approximately 30% of the land experienced robust moisture changes. The DIDWIW paradigm focuses on the moisture trends, which can capture their spatial patterns in detail. The two paradigms can be identified in the low precipitation-induced arid regions, which suggests that researchers consider these differences in the coupling of global climate and moisture trends.

## Methods

### Data source

The CCI global soil moisture data were obtained from the European Space Agency (ESA) (www.esa-soilmoisture-cci.org). They are fusion data that combine active and passive microwave satellite observations. The active data sets were generated by the University of Vienna and are based on observations from the C-band scatterometer on board the European Remote Sensing (ERS) satellites (ERS-1 and ERS-2) and the Meteorological Operational Satellite (MetOp-A). The passive data sets were generated by the VU University Amsterdam in collaboration with NASA and are based on observations from the Scanning Multichannel Microwave Radiometer (SMMR), the Special Sensor Microwave/Image (SSM/I), the Tropical Rainfall Measuring Mission microwave imager (TRMM TMI) and the Advanced Microwave Scanning Radiometer-Earth Observing System (AMSR-E).

The global precipitation data from the Global Precipitation Climatology Centre (GPCC) were obtained from (ftp://ftp-anon.dwd.de/pub/data/gpcc/html/download_gate.html). The Full Data Reanalysis (V.7 1901–2013) version was adopted from 1979 to 2013 for our study. The spatial resolution was resampled to 25 km to match the resolution of the CCI soil moisture data.

The global evapotranspiration data were calculated from the data set of latent heat flux provided by Jung *et al.* (https://www.bgc-jena.mpg.de/geodb/projects/Data.php). The flux data were generated by upscaling observations from the current global network of eddy-covariance towers^33^,^34^.

### Classification of the climate regions

The climate regions were classified based on the work of Koppen-Geiger. The classification captures the global wetness with a combination of the precipitation and air temperature (Table 1). Peel *et al.*^26^ updated the classification based on long-term monthly data sets of precipitation and temperature. We obtained the GRID climate map from (http://www.hydrol-earth-syst-sci.net/11/1633/2007/hess-11-1633- 2007-supplement.zip). The polar regions cover large areas of ice and snow, which were excluded in our study. Meanwhile, we defined and classified the arid regions with the first climate letter labelled Arid in Table 1, the humid regions with a second letter of *f* (without dry season) and the transitional regions as other climate zones^27^. The three climate regions (arid, humid and transitional) cover 30.39%, 42.83% and 26.78% of the global land area, respectively.

### Evaluations of the DGDWGW and DIDWIW paradigms

The DGDWGW paradigm implies that all arid and humid climate regions become drier and wetter, respectively. The agreement with the paradigm can be evaluated by the spatial consistency:


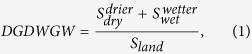


where 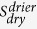 and 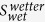
are areas of drier in dry and wetter in wet, respectively, and *S*_*land*_ is the global land area. If the paradigm was true (the trends are fully spatial consistent with the climate regions), then the calculated value would equal the area ratio of the arid and humid climate zones.

By contrast, the DIDWIW paradigm focuses on the spatial pattern of the moisture trend. We evaluated it using


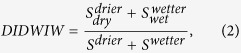


where *S*^*drier*^ and *S*^*wetter*^ are the areas of drier and wetter trends over land, respectively. A larger value indicates a higher reliability of the DIDWIW paradigm. The DIDWIW value reaches the maximum (equal to 1) when the whole drier and wetter trends occur in the arid and humid regions, respectively. The value equals 0 when none of the arid and humid regions become drier and wetter, respectively.

## Additional Information

**How to cite this article**: Feng, H. and Zhang, M. Global land moisture trends: drier in dry and wetter in wet over land. *Sci. Rep.* **5**, 18018; doi: 10.1038/srep18018 (2015).

## Figures and Tables

**Figure 1:**
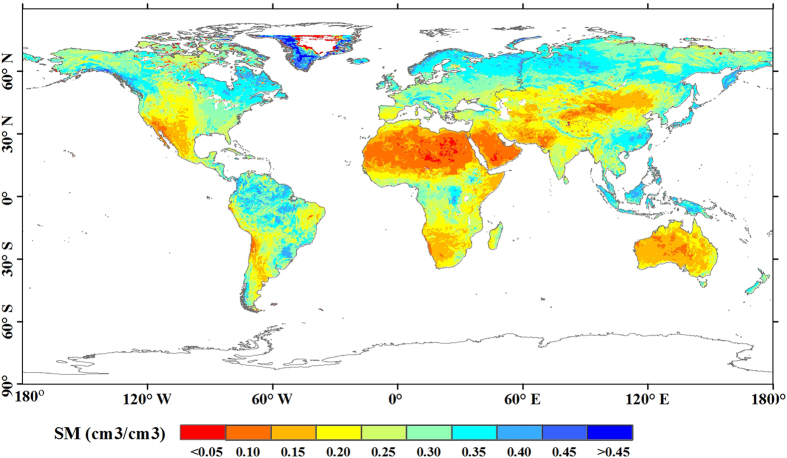
Multi-year mean of the global soil moisture from 1979 to 2013. The figure was generated using ArcGIS 10.0, and the coordinate system is the World Geodetic System 1984 (WGS84).

**Figure 2:**
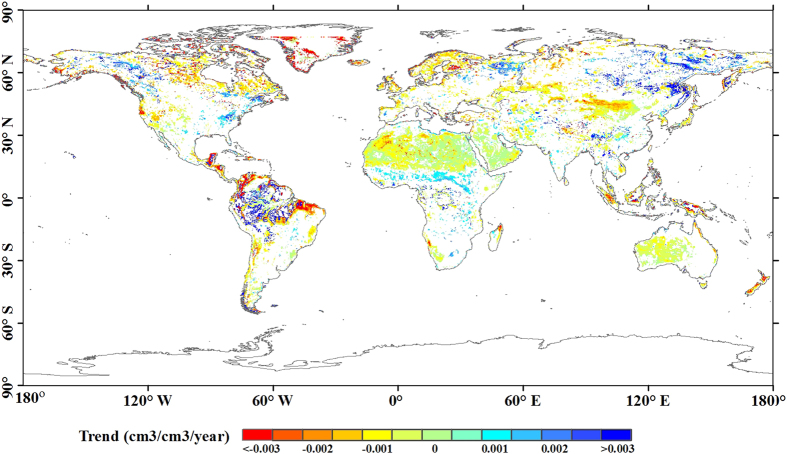
Temporal trends of the global soil moisture. Only the trends that are significant at p < 0.05 are presented. The figure was generated using ArcGIS 10.0, and the coordinate system is the World Geodetic System 1984 (WGS84).

**Figure 3:**
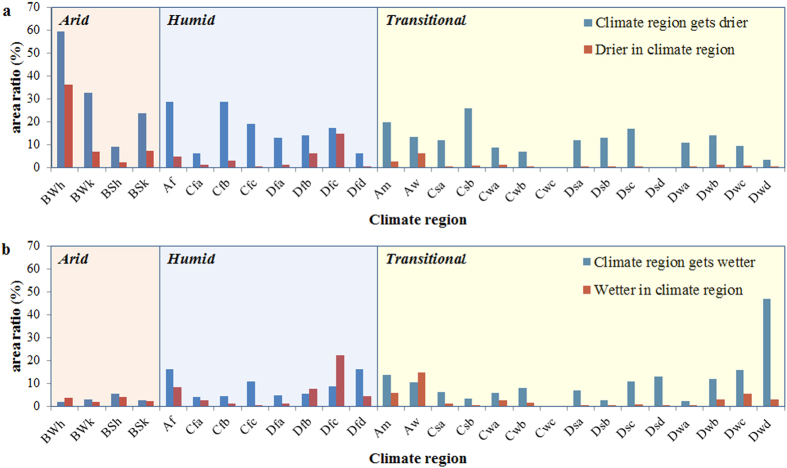
Statistics of the soil moisture trends. (**a**), The drier trend, and (**b**), the wetter trend. The abbreviations used to label the X-axis refer to the climate regions, which are composed of two or three letters. The first letter refers to the climate types: tropical (**A**), arid (**B**), temperate (**C**) and cold (**D**). The second letter indicates the precipitation conditions: rainforest (**f**), monsoon (**m**) and savannah (**s**) in tropical, desert (**W**) and steppe (**S**) in arid, dry summer (**s**), dry winter (**w**) and without dry season (**f**) in temperate and cold climates. The third letter designates hot (h) and cold (k) in arid and hot summer (**a**), warm summer (**b**), cold summer (**c**) and very cold summer (**d**) in temperate and cold climates. “Climate regions get drier/wetter” is the ratio of drier/wetter area for a climate region. “Drier/wetter in climate region” refers to the ratio of drier/wetter area in a climate region to the total drier/wetter area. The sum of “drier/wetter in climate region” equals 100%. The figure was generated using Microsoft Excel 2010.

**Table 1:** Description of the Koppen climate symbols and defining criteria (Peel *et al.*, 2007).

1^st^	2nd	3rd	Description	Criteria*
A			Tropical	T_cold_ ≥ 18
	f		- Rainforest	P_dry_ ≥ 60
	m		- Monsoon	Not (Af) & P_dry_ ≥ 100-MAP/25
	w		- Savannah	Not (Af) & P_dry_ < 100-MAP/25
B			Arid	MAP < 10 × P_threshold_
	W		- Desert	MAP < 5 × P_threshold_
	S		- Steppe	MAP ≥ 10 × P_threshold_
		h	- Hot	MAT ≥ 18
		k	- Cold	MAT < 18
C			Temperate	T_hot_ > 10 & 0 < T_cold_ < 18
	s		- Dry Summer	P_sdry_ < 40 & P_sdry_ < P_wwet_/3
	w		- Dry Winter	P_sdry_ < P_wwet_/10
	f		- Without dry season	Not (Cs) or (Cw)
		a	- Hot Summer	T_hot_ ≥ 22
		b	- Warm Summer	Not (a) & T_mon10_ ≥ 4
		c	- Cold Summer	Not (a or b) & 1 ≤ T_mon10_ < 4
D			Cold	T_hot_ > 10 & T_cold_ ≤ 0
	s		- Dry Summer	P_sdry_ < 40 & P_sdry_ < P_wwet_/3
	w		- Dry Winter	P_sdry_ < P_wwet/_10
	f		- Without dry season	Not (Ds) or (Dw)
		a	- Hot Summer	T_hot_ ≥ 22
		b	- Warm Summer	Not (a) & T_mon10_ ≥ 4
		c	- Cold Summer	Not (a, b or d)
		d	- Very Cold Winter	Not (a or b) & T_cold_ < -38
E			Polar	T_hot_ < 10
	T		- Tundra	T_hot_ > 0
	F		- Frost	T_hot_ ≤ 0

^*^The first letters (A to E) refer to the broad climate types. The second letters (f, m, w/W and s/S) are the subsequent precipitation conditions. Because no precipitation differentiations are given for the polar climates (E), the letters T and F are defined for the temperature conditions. The third letters are temperature classifications (h) and (k) for the arid climates (B) and (a) to (d) for the warm temperate and snow climates (C) and (D). MAP = mean annual precipitation, MAT = mean annual temperature, T_hot_ = temperature of the hottest month, T_cold_ = temperature of the coldest month, T_mon10_ = number of months where the temperature is above 10 °C, P_dry_ = precipitation of the driest month, P_sdry_ = precipitation of the driest month in summer, P_wdry_ = precipitation of the driest month in winter, P_swet_ = precipitation of the wettest month in summer, P_wwet_ = precipitation of the wettest month in winter, P_threshold_ = varies according to the following rules (if 70% of the MAP occurs in winter, then P_threshold_ = 2 × MAT; if 70% of the MAP occurs in summer, then P_threshold_ = 2 × MAT + 28, otherwise P_threshold_ = 2 × MAT + 14). Summer (winter) is defined as the warmer (cooler) six month period of ONDJFM and AMJJAS.
